# A Noninvasive, Economical, and Instant-Result Method to Diagnose and Monitor Type 2 Diabetes Using Pulse Wave: Case-Control Study

**DOI:** 10.2196/11959

**Published:** 2019-04-23

**Authors:** Yiming Hao, Feng Cheng, Minh Pham, Hayley Rein, Devashru Patel, Yuchen Fang, Yiyi Feng, Jin Yan, Xueyang Song, Haixia Yan, Yiqin Wang

**Affiliations:** 1 Shanghai Key Laboratory of Health Identification and Assessment/Laboratory of Traditional Chinese Medicine Four Diagnostic Information Shanghai University of Traditional Chinese Medicine Shanghai China; 2 Department of Pharmaceutical Sciences, College of Pharmacy University of South Florida Tampa, FL United States; 3 Department of Mathematics and Statistics, College of Arts and Sciences University of South Florida Tampa, FL United States; 4 Department of Cellular and Molecular Biology, College of Arts and Sciences University of South Florida Tampa, FL United States; 5 College of Computing Georgia Institute of Technology Atlanta, GA United States; 6 Jiangsu Province Hospital of Chinese Medicine Nanjing China

**Keywords:** type 2 diabetes, hypertension, hyperlipidemia, pulse wave analysis, diagnosis

## Abstract

**Background:**

We should pay more attention to the long-term monitoring and early warning of type 2 diabetes and its complications. The traditional blood glucose tests are traumatic and cannot effectively monitor the development of diabetic complications. The development of mobile health is changing rapidly. Therefore, we are interested in developing a new noninvasive, economical, and instant-result method to accurately diagnose and monitor type 2 diabetes and its complications.

**Objective:**

We aimed to determine whether type 2 diabetes and its complications, including hypertension and hyperlipidemia, could be diagnosed and monitored by using pulse wave.

**Methods:**

We collected the pulse wave parameters from 50 healthy people, 139 diabetic patients without hypertension and hyperlipidemia, 133 diabetic patients with hypertension, 70 diabetic patients with hyperlipidemia, and 75 diabetic patients with hypertension and hyperlipidemia. The pulse wave parameters showing significant differences among these groups were identified. Various machine learning models such as linear discriminant analysis, support vector machines (SVMs), and random forests were applied to classify the control group, diabetic patients, and diabetic patients with complications.

**Results:**

There were significant differences in several pulse wave parameters among the 5 groups. The parameters height of tidal wave (h_3_), time distance between the start point of pulse wave and dominant wave (t_1_), and width of percussion wave in its one-third height position (W) increase and the height of dicrotic wave (h_5_) decreases when people develop diabetes. The parameters height of dominant wave (h_1_), h_3_, and height of dicrotic notch (h_4_) are found to be higher in diabetic patients with hypertension, whereas h_5_ is lower in diabetic patients with hyperlipidemia. For detecting diabetes, the method with the highest out-of-sample prediction accuracy is SVM with polynomial kernel. The algorithm can detect diabetes with 96.35% accuracy. However, all the algorithms have a low accuracy when predicting diabetic patients with hypertension and hyperlipidemia (below 70%).

**Conclusions:**

The results demonstrated that the noninvasive and convenient pulse-taking diagnosis described in this paper has the potential to become a low-cost and accurate method to monitor the development of diabetes. We are collecting more data to improve the accuracy for detecting hypertension and hyperlipidemia among diabetic patients. Mobile devices such as sport bands, smart watches, and other diagnostic tools are being developed based on the pulse wave method to improve the diagnosis and monitoring of diabetes, hypertension, and hyperlipidemia.

## Introduction

### Background

Diabetes is becoming one of the most severe health problems in China. The World Health Organization indicated that type 2 diabetes accounts for around 90% of all the diabetes cases worldwide. In 2016, 9.4% of the population of China was diabetic, and 2% of all deaths in the country were because of diabetes and its chronic complications [1,2]. Type 2 diabetes and a variety of chronic complications in the middle and late stages of the disease develop over a long time. Therefore, early diagnosis, prevention, and treatment of type 2 diabetes are vital for reducing the medical burden and mortality rate. Diabetes is a long-standing example of a disease whose patients have been positively impacted by traditional Chinese medicine (TCM) [3]. Accurate diagnosis through the noninvasive, convenient, and economical techniques of TCM allows effective prevention and treatment of type 2 diabetes.

In TCM diagnosis, pulse taking is an important skill for diagnosing diseases by touching and sensing radial pulsations, but it frequently depends on the subjective consciousness and experience accumulation of the doctors. It is the lack of objective criteria that reduces the accuracy and repeatability of diagnosis. To overcome the shortcomings of subjectivity of traditional pulse diagnosis, in recent years, the importance of objective pulse-taking diagnosis has gained more and more attention. In addition, the study of diagnosis of some chronic diseases such as coronary heart disease and lung cancer by using the objective pulse parameters had some progress [4,5]. In the study of type 2 diabetes, some researchers have found that the changes of radial artery pulse wave are related to elevated blood glucose levels and major adverse cardiovascular events caused by diabetes mellitus [6,7]. Therefore, we hope that the noninvasive, convenient, and objective pulse information detection can be used to assist conventional methods to diagnose and monitor the occurrence and development of type 2 diabetes.

### Objectives

The purpose of this study was to use a *TCM pulse informatics analysis system* to measure people’s objective pulse information and use that data to develop a model to diagnose type 2 diabetes. This instrument uses a pressure sensor to record pulse beats and display this information within a pulse wave. The pressure sensor is the most commonly used device for recording pulse wave of people’s radial artery at present. The parameters of the pulse wave are extracted and analyzed using statistical methods. This approach can quantitatively analyze pulse signals and provide more objective results than traditional pulse-taking diagnosis by TCM doctors.

This is the first study to use this method to analyze the differences in pulse wave parameters between healthy individuals and diabetic patients with hypertension and hyperlipidemia. The purpose of our research was (1) to find the association of the objective pulse information with type 2 diabetes and the disease with hypertension or hyperlipidemia and (2) to establish the prediction models of diabetes and its complications. We hypothesized that diabetes and its combination with hypertension and hyperlipidemia can be diagnosed reliably by using the pulse wave parameters.

## Methods

### Patients

This is a case-control study. Patients with type 2 diabetes (referred to as diabetes from now on) were recruited from outpatient services in 4 hospitals in Shanghai, including Yueyang Hospital of Integrated Traditional Chinese and Western Medicine, Shuguang Hospital, Shanghai Municipal Hospital of TCM, and Shanghai Qiangsheng Worker’s Hospital, between April 2012 and December 2018. The individuals in the healthy control group (group 1, n=50) were healthy staff and graduate students from the Shanghai University of TCM. The subjects were of Han ethnicity.

On the basis of blood pressure and serum lipid levels, diabetic patients were divided into 4 groups, including diabetic patients without hypertension and hyperlipidemia (group 2, n=139), diabetic patients with hypertension (group 3, n=133), diabetic patients with hyperlipidemia (group 4, n=70), and diabetic patients with hypertension and hyperlipidemia (group 5, n=75).

There was no significant difference in the age (*P=*.13) or gender (*P=*.59) between healthy control individuals (group 1) and all diabetic patients (group 2-5) (Table 1).

**Table 1:** Summary of demographics and clinic characteristics of each group.

Group	Cases	Ratio of male to female	Average age (years)	Average course of disease (years)
Group 1	50	1:1.27	61.40 (SD 10.08)	NA^a^
Group 2-5	417	1:1.40	63.67 (SD 11.15)	7.36 (SD 6.44)
Group 2	139	1:1.62	61.10 (SD 11.34)	6.28 (SD 5.99)
Group 3	133	1:1.15	67.97 (SD 10.22)	8.04 (SD 6.44)
Group 4	70	1:1.26	59.20 (SD 11.29)	6.22 (SD 5.26)
Group 5	75	1:1.68	64.96 (SD 9.37)	8.63 (SD 7.55)

^a^NA: not applicable.

### Ethics Approval

The study was approved by the Ethics Committee of Shanghai University of TCM in China in January 2012 and performed in accordance with the Declaration of Helsinki. All subjects had signed informed consent agreements.

### Criteria

#### Diagnostic Criteria

The diagnostic criteria of diabetes were referred to as the *Standards of medical care in diabetes* [8].

#### Inclusion Criteria

The inclusion criteria were as follows:

1. Meet the diagnostic standard of diabetes
2. Age range is from 40 to 75 years
3. Hypertension or hyperlipidemia occur after diabetes.

#### Exclusion Criteria

The exclusion criteria were as follows:

1. Women during their pregnancy and lactation period
2. Mentally ill individuals
3. Those complicated with other diseases
4. Individuals who had acute metabolic disorders such as diabetic ketoacidosis or inflammatory complications.

#### Elimination Criteria

Patients without complete clinical data either because of incomplete collection or missing data were eliminated.

### Collecting Methods for the Objective Parameters of Traditional Chinese Medicine Diagnosis

**Figure 1:**
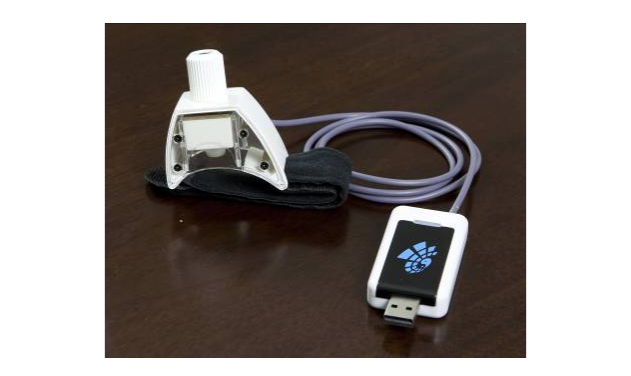
Traditional Chinese medicine pulse life informatics analysis system.

**Figure 2:**
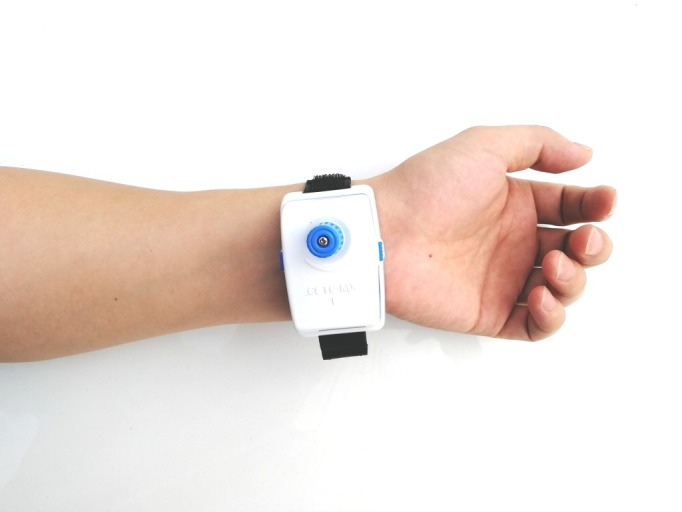
The wristband pulse wave information acquisition terminal.

**Figure 3:**
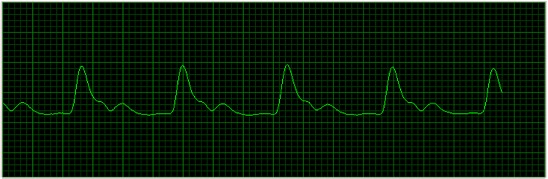
The pulse wave image collected.

All objective parameters of TCM pulse diagnosis were collected by 2 MD doctors using the TCM pulse informatics analysis system (type: Smart TCM-I, product by: Shanghai Asia & Pacific Computer Information System CO, Ltd, Shanghai, China; Figure 1). In the analysis system, the instrument of detecting pulse wave information is a wristband acquisition terminal (Figure 2). The device consists of a pulse sensor, adapter, and acquisition software. The pulse sensor is attached to the arm by a wristband and connected to the adapter by a cable. The adapter has a universal serial bus (USB) connector, which can be directly connected to the computer through the USB interface. The acquisition software runs on the computer and realizes the acquisition and data management function of the pulse wave image by cooperating with the hardware (Figure 3).

The indoor temperature was 18°C to 25°C during pulse wave information collection. The subjects were either sitting or lying down for at least 3 min before they were tested. For pulse-taking diagnosis data collection, the forearms of the patients were extended forward naturally and at the same height as the heart. Their wrists were kept straight, with palms upward and fingers slightly bent. A small, soft pillow was placed under the wrist joint for better data collection. The pulse was measured at the radial artery corresponding to the inside of the styloid process of the radius in the left hand, the best position to feel the pulse. The frequency of the pulse acquisition device was 720 Hz. The sensor was tied to the position of the radial artery, and the pressure was adjusted by a knob. The acquisition was realized through observation and operation on the software interface. The software interface displayed the dynamic pulse waveform and pressure value in real time. The software recorded the pulse wave parameters for 1 min when the amplitude of the pulse wave reaches the maximum value. Afterward, the parameters of the pulse wave were extracted through the built-in software.

**Figure 4:**
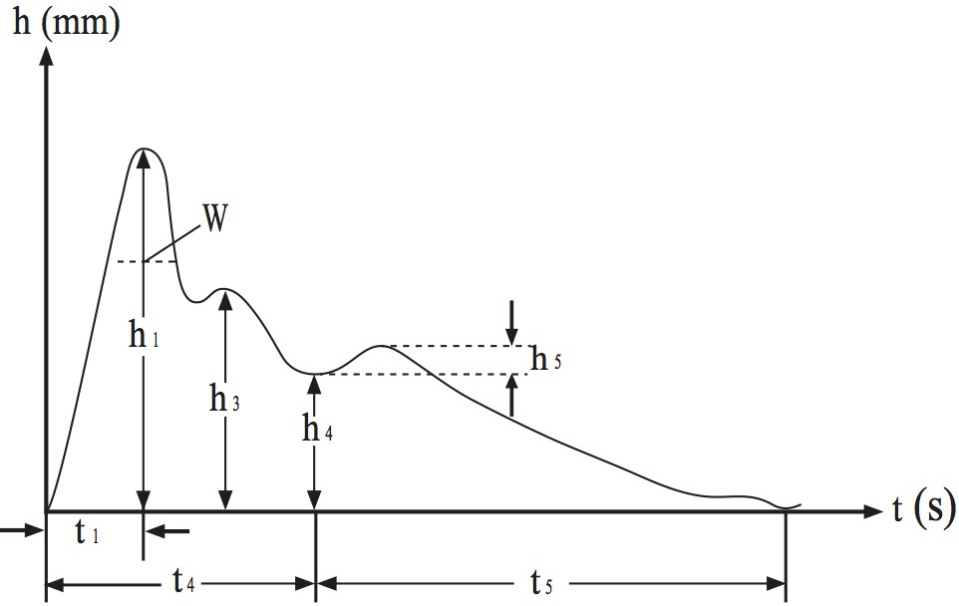
A typical single pulse wave. h: height; h_1_: height of dominant wave; h_3_: height of tidal wave; h_4_: height of dicrotic notch; h_5_: height of dicrotic wave; mm: millimeter; t: time; s: second; t_1_: time distance between the start point of pulse wave and dominant wave; t_4_: time distance between the start point of pulse wave and dicrotic notch; t_5_: time distance between dicrotic notch and the end point of pulse wave; W: width of percussion wave in its one-third height position.

The data collected could be presented as a time-domain pulse wave (Figure 4), which includes the height of dominant wave (h_1_), height of tidal wave (h_3_), height of dicrotic notch (h_4_), height of dicrotic wave (h_5_), time distance between the start point of pulse wave and dominant wave (t_1_), time distance between the start point of pulse wave and dicrotic notch (t_4_), time distance between dicrotic notch and the end point of pulse wave (t_5_), and width of percussion wave in its one-third height position (W).

The pulse wave parameters, including h_1_, h_3_, h_4_, h_5_, t_1_, t_4_, t_5_, and W, have different physiological significances (Table 2).

### Disease Prediction Methods and Statistical Analysis

The first objective was to analyze the differences in pulse wave parameters between healthy individuals and diabetic patients, and among the 4 subgroups of diabetic patients. One-way analysis of variance (ANOVA) was applied to the pulse wave parameters that follow the normal distribution, and non-parametric methods including the Mann-Whitney U test (for 2 groups) and the Kruskal-Wallis one-way ANOVA test (for 4 groups) were used for non-normal data.

The second objective was to classify diabetic patients from healthy individuals and to detect hypertension and hyperlipidemia among diabetic patients by using the pulse wave parameters. The following machine learning algorithms were applied:

- Logistic regression using all the pulse wave parameters as predictors
- Linear discriminant analysis (LDA) using all the pulse wave parameters as predictors
- Random forests with 500 decision trees, each tree samples two-thirds of the data, and each split in a decision tree randomly samples 3 out of 11 pulse wave parameters.
- Support vector machines (SVMs): We applied SVM with linear kernel (SVM-Linear) and SVM with 3-degree polynomial kernel (SVM-Poly). The cost and gamma parameters are determined through cross-validation, with trial values for cost being 0.001, 0.01, 0.1, 1, 5, 10, and 100 and trial values for gamma being 0.001, 0.01, and 0.1.

To estimate the prediction accuracy of the models (out-of-sample accuracy), we applied the 10-fold cross-validation technique.

**Table 2:** Physiological significance of pulse wave parameters.

Pulse wave parameters		Physiological significance
**Height parameters of pulse wave**		
	Height of dominant wave (h_1_)	High value of h_1_ reflects the strong elasticity of large artery and good ejection function of the left ventricle
	Height of tidal wave (h_3_)	High value of h_3_ reflects the weak elasticity and/or high peripheral resistance of the artery
	Height of dicrotic notch (h_4_)	High value of h_4_ reflects the high diastolic blood pressure, high peripheral resistance of the artery, and/or weak closing function of the aortic valve
	Height of dicrotic wave (h_5_)	High value of h_5_ reflects the strong elasticity of the large artery and good closing function of the aortic valve
**Time parameters of pulse phase**		
	Time distance between the start point of pulse wave and dominant wave (t_1_)	High value of t_1_ reflects the weak elasticity of large vessels and/or high tensioning of small vessels
	Time distance between the start point of pulse wave and dicrotic notch (t_4_)	High value of t_4_ reflects the good systolic function of the heart
	Time distance between dicrotic notch and the end point of pulse wave (t_5_)	High value of t_5_ reflects the weak elasticity and/or high peripheral resistance of large vessels
	Width of percussion wave in its one-third height position (W)	High value of W reflects the long time maintained by the high pressure in the artery

## Results

### Difference in Pulse Wave Parameters Between Groups

Tables 3 and 4 show the ANOVA test and nonparametric test results on the pulse wave parameters between groups.

The results indicated that all diabetic patients (groups 2-5) had significantly higher h_3_, t_1_, and W than those in the healthy control group, whereas h_5_ was significantly lower in diabetics when compared with healthy individuals. The parameters of pulse wave height, h_1_, h_3_, h_4_, and h_5,_ were significantly different in groups 2 to 5. On the contrary, there were no significant differences in parameters of time distance, t_1_, t_4_, t_5_, and W, among these groups. Pairwise comparisons indicated that the h_1_ values of diabetic patients with hypertension (groups 3 and 5) were significantly higher than those of diabetic patients with hyperlipidemia (group 4). In addition, the h_1_ values of diabetic patients with hypertension (group 3) were also significantly higher than those of diabetic patients without hypertension and hyperlipidemia (group 2). Diabetic patients without hypertension (groups 2 and 4) showed a significantly lower h_3_ parameter than those of diabetic patients with hypertension (groups 3 and 5). The h_4_ parameter of group 2 was significantly lower than that of groups 5. Similar to h_1_ and h_3_, h_4_ values of groups 3 and 5 were also significantly higher than those of group 4. The value of parameter h_5_ in group 5 was significantly lower than that of parameter h_5_ in groups 2, 3, and 4.

**Table 3:** Comparison of pulse wave parameters between group 1 and group 2-5.

Parameter	Group 1	Groups 2-5	*P* value
Height of dominant wave (h_1_) (mm)	11.58 (9.65-13.68)	11.98 (8.25-16.60)	.48
Height of tidal wave (h_3_) (mm)	7.74 (6.54-9.96)	9.83 (6.71-13.95)	.001
Height of dicrotic notch (h_4_) (mm)	4.69 (4.24-6.25)	5.47 (3.32-7.36)	.44
Height of dicrotic wave (h_5_) (mm)	5.48 (4.66-6.70)	2.48 (0.24-5.00)	<.001
Time distance between the start point of pulse wave and dominant wave (t_1_) (s)	0.11 (0.10-0.12)	0.14 (0.12-0.18)	<.001
Time distance between the start point of pulse wave and dicrotic notch (t_4_) (s)	0.33 (0.30-0.35)	0.33 (0.31-0.36)	.24
Time distance between dicrotic notch and the end point of pulse wave (t_5_) (s)	0.45 (0.42-0.54)	0.49 (0.42-0.59)	.15
Width of percussion wave in its one-third height position (W) (s)	0.17 (0.13-0.22)	0.21 (0.18-0.23)	<.001

**Table 4:** Comparison of pulse wave parameters between groups 2, 3, 4, and 5.

Parameter	Group 2	Group 3	Group 4	Group 5	*P* value
Height of dominant wave (h_1_) (mm)	11.56 (8.07-16.17)	13.04 (9.82-18.21)^a^	10.80 (7.39-14.22)^b^	12.65 (8.50-18.77)^c^	.01
Height of tidal wave (h_3_) (mm)	8.87 (6.55-13.35)	10.89 (7.33-14.99)^a^	8.98 (6.02-12.18)^b^	10.74 (7.78-15.42)^b,c^	.01
Height of dicrotic notch (h_4_) (mm)	4.66 (3.29-6.97)	5.84 (3.48-7.77)	4.78 (2.65-6.68)^b^	5.87 (3.70-8.60)^a^^□^	.02
Height of dicrotic wave (h_5_) (mm)	2.86 (0.88-5.05)	2.80 (0.22-5.60)	2.42 (1.00-4.51)	1.17 (-0.60-3.85)^a,b,c^	.004
Time distance between the start point of pulse wave and dominant wave (t_1_) (s)	0.14 (0.12-0.18)	0.14 (0.12-0.18)	0.14 (0.12-0.19)	0.14 (0.12-0.16)	.66
Time distance between the start point of pulse wave and dicrotic notch (t_4_) (s)	0.33 (0.31-0.35)	0.33 (0.30-0.36)	0.33 (0.31-0.35)	0.33 (0.32-0.36)	.67
Time distance between dicrotic notch and the end point of pulse wave (t_5_) (s)	0.48 (0.41-0.60)	0.48 (0.42-0.61)	0.51 (0.43-0.59)	0.49 (0.43-0.55)	.87
Width of percussion wave in its one-third height position (W) (s)	0.21 (0.18-0.23)	0.21 (0.18-0.24)	0.21 (0.18-0.24)	0.21 (0.18-0.22)	.87

^a^Means compared with group 2; *P*<.05.

^b^Means compared with group 3; *P*<.05.

^c^Means compared with group 4; *P*<.05.

**Table 5:** Physiological significance of pulse wave parameters.

Method	Accuracy to detect diabetes	Accuracy to detect hypertension	Accuracy to detect hyperlipidemia
Logistic regression	0.9293	0.5920	0.6500
Linear discriminant analysis	0.9037	0.5944	0.6500
Random forests	0.9294	0.5697	0.6977
SVM^a^ with linear kernel	0.9421	0.5780	0.6572
SVM with polynomial kernel	0.9635	0.5858	0.6821

^a^SVM: support vector machine.

**Table 6:** Algorithm statistics.

Diagnosis of	Method used	Accuracy	Sensitivity	Specificity
Diabetes	SVM^a^ with polynomial kernel	0.9635	0.8571	0.9535
Hypertension	Linear discriminant analysis	0.5944	0.7419	0.5429
Hyperlipidemia	Random forests	0.6977	0.7333	0.6190

^a^SVM: support vector machine.

### Classification Algorithms

The accuracies of the classification algorithms are presented in Table 5.

Table 4 indicates that SVM with polynomial kernel (tuned parameters are cost=100 and gamma=0.01) has the highest prediction accuracy for diabetes, LDA has the highest prediction accuracy for hypertension among diabetic patients, and random forests has the highest prediction accuracy for hyperlipidemia among diabetic patients. The maximum accuracy, sensitivity, and specificity for each diagnosing task are presented in Table 6.

## Discussion

### Principal Findings

Pulse wave is the track of radial artery pulsation. It integrates a large amount of useful information about heart ejection activity and how the pulse wave travels along the vascular tree [9]. In this paper, time-domain analysis, one of the most frequently used techniques for pulse research [9], was applied to study the association between pulse parameters (including the height of pulse wave and the time of pulse phase) and type 2 diabetes to predict the attack of type 2 diabetes and its complications (hypertension and hyperlipidemia) for the first time.

Hyperglycemia is a leading cause of cardiovascular disease [10]. Therefore, the incidence of atherosclerosis is increased 2 to 4 folds in diabetic patients compared with nondiabetic individuals [11]. These diabetic patients experience hardened arterial blood vessels and decreased arterial elasticity, which increases peripheral resistance and the duration of high pressure in blood vessels. In this paper, we found that the parameters of h_3_, t_1_, and W increased significantly in diabetic patients compared with healthy individuals. From the result, we know that the high value of h_3_ gives rise to weak elasticity and high peripheral resistance of arterial blood vessels. The high t_1_ value reflects the weak elasticity of large vessels and high tensioning of small vessels, and the high W value denotes that high pressure in the artery is maintained for a longer period. Diversely, the value of h_5_ was significantly lower in diabetic patients when compared with their healthy counterparts. The high h_5_ value reflects the strong elasticity of the large artery. Our results from pulse time-domain analysis agreed well with the findings on patients and rats with diabetes [12,13].

Our analysis revealed that diabetic patients with hyperlipidemia had lower h_5_ values compared with patients without hyperlipidemia, indicating that the arterial blood vessels were more rigid and less elastic in hyperlipidemia patients. These results are consistent with previous findings of high arteriosclerosis incidence and reduced hemodynamic functions in hyperlipidemia patients [14].

Our results also showed that h_1_, h_3_, and h_4_ were relatively high in diabetic patients with hypertension compared with patients without hypertension, which can be explained by elevated arterial pressure because of high systolic and diastolic blood pressure in hypertension patients.

The prediction accuracy for diabetes is 96.35% (SVM-Poly). The algorithm can be used in mobile devices to conveniently and reliably diagnose diabetes. Predicting hypertension and hyperlipidemia among diabetic patients has low accuracy, 59.44% (LDA) and 69.77% (random forests), respectively. This may be explained by 2 reasons. First, the sample size for classifying hypertension and hyperlipidemia is relatively small (n=417). Second, hyperglycemia, hypertension, and hyperlipidemia can aggravate the microvascular and macrovascular lesions in diabetic patients [15], such as the decrease in vascular elasticity and the increase of intravascular pressure. Therefore, the differences between diabetic patients with hypertension/hyperlipidemia and patients without hypertension/hyperlipidemia may not be obvious. We plan to collect more samples, and we hope the accuracy of the models for identifying diabetic patients with hypertension/ hyperlipidemia may increase with the larger sample size.

### Strengths and Limitations

With the growing maturity of sensors, chips, mobile internet, and other technologies, people’s health awareness is increasing and the demand for health services is greatly improved. The demand for wearable technology is becoming more and more high. Wearable and portable devices have shown great potential in the field of medical health. Pulse-taking diagnosis has several advantages such as its noninvasive nature and convenience. In our research, the pulse wave information acquisition element is a pressure sensor, which is the most commonly used device. Although there is another new type of sensor called an *ultrasonic sensor* used to detect pulse, the traditional pressure sensor is more cost-effective. In recent years, mobile phone apps are becoming more and more useful for the self-management of diabetes [16-18]. The wristband acquisition terminal we used also provides the possibility of integrating pulse parameter collection to mobile devices (such as sport bands and watches) and data analysis through mobile apps in the future.

The results from our study have confirmed the findings from previous studies [10-15]. Our methods showed a very high diagnostic accuracy for type 2 diabetes, whereas the diagnosis of hypertension and hyperlipidemia is not yet reliable. If we continuously improve the accuracy of using objective pulse information to detect type 2 diabetes in future research, the noninvasive and convenient pulse-taking diagnosis technique may have the potential to become a low-cost and accurate method to help people monitor the occurrence and development of type 2 diabetes and its complications more conveniently in daily life. It is important to note that in our research, when we used this device to collect pulse wave information, the subjects were asked to keep quiet and relaxed to measure the pulsation of the radial artery of their left hands under the appropriate temperature (as mentioned in the section Collecting Methods for the Objective Parameters of Traditional Chinese Medicine Diagnosis). Therefore any mobile device that uses our model will rely on the user being able to replicate the same condition. In the future, we hope to improve the convenience and stability of the device so that people can use it on both the left and right hands when moving. We are currently developing such devices and programs for the improvement of diabetes monitoring and remote diagnosis.

## Abbreviations

ANOVA: analysis of variance
h_1_: height of dominant wave
h_3_: height of tidal wave
h_4_: height of dicrotic notch
h_5_: height of dicrotic wave
LDA: linear discriminant analysis
SVM: support vector machine
TCM: traditional Chinese medicine
t_1_: time distance between the start point of pulse wave and dominant wave
t_4_: time distance between the start point of pulse wave and dicrotic notch
t_5_: time distance between dicrotic notch and the end point of pulse wave
USB: universal serial bus
W: width of percussion wave in its one-third height position

## References

[1] (2016). World Health Organization.

[2] (2016). World Health Organization.

[3] Xu M (2010). Diabetes mellitus (Second Edition).

[4] Wang Y, Xu J, Guo R, Xu C, Hao Y, Chen C, Hong Y, Xiao X, Xu W, Hong J, Lei Z (2014). Therapeutic effect in patients with coronary heart disease based on information analysis from traditional Chinese medicine four diagnostic methods. J Tradit Chin Med.

[5] Zhang Z, Zhang Y, Yao L, Song H, Kos A (2018). A sensor-based wrist pulse signal processing and lung cancer recognition. J Biomed Inform.

[6] Zhang M, Bai Y, Ye P, Luo L, Xiao W, Wu H, Liu D (2011). Type 2 diabetes is associated with increased pulse wave velocity measured at different sites of the arterial system but not augmentation index in a Chinese population. Clin Cardiol.

[7] Chang C, Liao K, Chang Y, Wang S, Chen Y, Wang G (2019). The effect of radial pulse spectrum on the risk of major adverse cardiovascular events in patients with type 2 diabetes. J Diabetes Complications.

[8] American Diabetes Association (2014). Standards of medical care in diabetes--2014. Diabetes Care.

[9] Fei Z (1991). Study on pulse-taking in China.

[10] Roussel R, Steg PG, Mohammedi K, Marre M, Potier L (2018). Prevention of cardiovascular disease through reduction of glycaemic exposure in type 2 diabetes: a perspective on glucose-lowering interventions. Diabetes Obes Metab.

[11] Khaleeli E, Peters S, Bobrowsky K, Oudiz R, Ko J, Budoff M (2001). Diabetes and the associated incidence of subclinical atherosclerosis and coronary artery disease: implications for management. Am Heart J.

[12] Harper E, Forde H, Davenport C, Rochfort KD, Smith D, Cummins PM (2016). Vascular calcification in type-2 diabetes and cardiovascular disease: integrative roles for OPG, RANKL and TRAIL. Vascul Pharmacol.

[13] Li T, Ni L, Liu X, Wang Z, Liu C (2016). High glucose induces the expression of osteopontin in blood vessels in vitro and in vivo. Biochem Biophys Res Commun.

[14] Sharma A, Tate M, Mathew G, Vince JE, Ritchie RH, de Haan JB (2018). Oxidative stress and NLRP3-inflammasome activity as significant drivers of diabetic cardiovascular complications: therapeutic implications. Front Physiol.

[15] Khunti K, Kosiborod M, Ray K (2018). Legacy benefits of blood glucose, blood pressure and lipid control in individuals with diabetes and cardiovascular disease: Time to overcome multifactorial therapeutic inertia?. Diabetes Obes Metab.

[16] Desveaux L, Shaw J, Saragosa M, Soobiah C, Marani H, Hensel J, Agarwal P, Onabajo N, Bhatia RS, Jeffs L (2018). A mobile app to improve self-management of individuals with type 2 diabetes: qualitative realist evaluation. J Med Internet Res.

[17] Lunde P, Nilsson BB, Bergland A, Kværner KJ, Bye A (2018). The effectiveness of smartphone apps for lifestyle improvement in noncommunicable diseases: systematic review and meta-analyses. J Med Internet Res.

[18] Hou C, Xu Q, Diao S, Hewitt J, Li J, Carter B (2018). Mobile phone applications and self-management of diabetes: a systematic review with meta-analysis, meta-regression of 21 randomized trials and GRADE. Diabetes Obes Metab.

